# Effects of *Lactobacillus Fermentum* Supplementation on Body Weight and Pro-Inflammatory Cytokine Expression in *Campylobacter Jejuni*-Challenged Chickens

**DOI:** 10.3390/vetsci7030121

**Published:** 2020-08-29

**Authors:** Miroslava Šefcová, Marco Larrea-Álvarez, César Larrea-Álvarez, Viera Revajová, Viera Karaffová, Jana Koščová, Radomíra Nemcová, David Ortega-Paredes, Christian Vinueza-Burgos, Mikuláš Levkut, Róbert Herich

**Affiliations:** 1 Department of Pathological Anatomy and Pathological Physiology, University of Veterinary Medicine and Pharmacy, Komenského 73, 040 01 Košice, Slovakia; viera.revajova@uvlf.sk (V.R.); viera.karaffova@uvlf.sk (V.K.); mikulas.levkut@uvlf.sk (M.L.); robert.herich@uvlf.sk (R.H.); 2 School of Biological Sciences and Engineering, Yachay-Tech University Hacienda San José, Imbabura, Urcuquí 100650, Ecuador; malarrea@yachaytech.edu.ec; 3 Research Unit, Life Science Initiative (LSI), lsi-ec.com, Quito 170102, Ecuador; cmla88@hotmail.com (C.L.-Á.); daortegap@gmail.com (D.O.-P.); 4 Department of Microbiology and Immunology, University of Veterinary Medicine and Pharmacy, Komenského 73, 040 01 Košice, Slovakia; jana.koscova@uvlf.sk (J.K.); radomira.nemcova@uvlf.sk (R.N.); 5 Facultad de Medicina Veterinaria y Zootecnia, Unidad de Investigación en Enfermedades Transmitidas por Alimentos y Resistencia a los Antimicrobianos (UNIETAR), Universidad Central del Ecuador, Quito 170129, Ecuador; cvinueza@uce.edu.ec; 6 Institute of Neuroimmunology, Slovak Academy of Science, Dúbravská cesta 5779/9, 84510 Bratislava, Slovakia

**Keywords:** broiler chicken, *Lactobacillus fermentum*, *Campylobacter jejuni*, cytokine gene expression, body weight

## Abstract

Due to the interest in using probiotic bacteria in poultry production, this research was focused on evaluating the effects of *Lactobacillus fermentum* Biocenol CCM 7514 administration on body weight gain and cytokine gene expression in chickens challenged with *Campylobacter jejuni*. One-hundred and eight 1-day old COBB 500 broiler chickens were equally assigned to four experimental groups at random. In the control group (C) chicks were left untreated, whereas in groups LB and LBCj a suspension of *L. fermentum* was administered. A suspension of *C. jejuni* was subsequently applied to groups Cj and LBCj. Body weight was registered, and the individuals were later slaughtered; cecum samples were collected at 12, 36 and 48 h post-infection (hpi). The entire experiment lasted seven days. Reverse transcription quantitative PCR (RT-qPCR) was used to determine expression levels of IL-1β, IL-15, IL-17, and IL-18 at each time point. Pathogen-infected individuals were observed to weigh significantly less than those fed with the probiotic. Significant differences were also found in transcript abundance; expression of IL-15 was downregulated by the probiotic and upregulated by *C. jejuni*. The effects of bacterial treatments were time-dependent, as the expression profiles differed at later stages. The present outcomes demonstrate that *L. fermentum* both reduces the impact of *C. jejuni* infection on chicken body weight and regulates positively pro-inflammatory cytokine expression, which ultimately increase bird well-being and improves production.

## 1. Introduction

*Campylobacter jejuni* has been considered one of the leading causes of human gastrointestinal diseases worldwide, with outbreaks registered both in industrialized and developing countries [1]. *Campylobacter* spp. colonizes the avian gut in high concentrations with few or no clinical symptoms. Hence, it has been traditionally considered commensal, although a revision of this bacteria–host interaction has been recently proposed [2]. Upon interaction with avian epithelial cells, *C. jejuni* stimulates the expression of various pro-inflammatory cytokines (TNF-α, IL-1β, IFN-γ, IL-6, and IL-2). Such conditions can lead to weight loss, intestinal damage and potential neurological disease, along with significant economic losses for poultry productivity [3,4]. The host immune response and the outcome of a *C. jejuni* colonization have been observed to be highly influenced by many factors, including chicken breed. It has been demonstrated that broiler-type birds mount a more vigorous response than layer-type birds [5]. However, some broiler lines have proved to be more susceptible than others to a *C. jejuni* infection, which is nonetheless characterized by an extended inflammatory response and an induction of lymphocyte activation [6,7]. Fast-growing broiler breeds, such as COBB 500, are widely used commercially. Several investigations have shown that this breed of broiler does indeed mount an important immune response against *Campylobacter* colonization [8,9,10,11]

Excessive use of antibiotics in food animals (e.g., fluoroquinolones) is not only known to promote the selection of resistant bacterial strains [12,13], but also to cause dysbiosis in chickens, which ultimately leads to a long-term diminished immune reaction [14,15]. The concept of early life programming, which assumes that the development of diseases later in life can be modulated by environmental exposures during critical pre- or early post-natal life, has recently arisen as particularly relevant because broilers are selected for rapid early growth, so the development of their immune system occurs mainly early in life [16]. For instance, chickens inoculated with three species of *Lactobacillus* (*L. ingluviei, L. agilis,* and *L. reuteri*), immediately post hatching, showed a significant increment in weight by 28 d of age. Furthermore, a reduction of pathogenic taxa such as the *Shigella* and *Escherichia* species was observed [17]. In addition, application of probiotics is known to promote an overall downregulation of pro-inflammatory mediators, including as IL-1β, TLR2, and TLR4 [18]. In particular, some species of *Lactobacillus* have proven useful for reducing the extent of *C. jejuni* colonization [19]. It has been demonstrated that early colonization of the gut (4 days of age) by *Campylobacter spp.* elicits an important immune reaction, including the regulation of various pro-inflammatory cytokines. However, this reaction can be significantly modulated by early treatment with probiotics [8,9,10,20]. IL-1β and IL-18 have been described as important players in the response to a *C. jejuni* infection in the presence of *L. reuteri* or *L. salivarius* [8,19]. These cytokines are important mediators in the initiation of inflammation, as their activation is known to increase the capacity of the innate immune system to react more efficiently against bacterial infections [21]. Various strains of *L. fermentum* have been regarded as promising probiotics, and have been suggested as potential tools for fighting *C. jejuni* infections, although these results come mainly from in vitro experiments [22,23]. Recently, we demonstrated that treatment with *L. fermentum* Biocenol CCM 7514 in chickens infected with *C. coli* promoted a heightened humoral response while dampening potential inflammation mediated by effector T cells in 1-week-old-chickens [20]. This study also revealed that the downregulation of IL-15 and IL-17, promoted by the probiotic, might be responsible for maintaining intraepithelial lymphocytes in an induced state and avoid their differentiation into effector cells. Moreover, early colonization by *C. jejuni* is known to induce a decrease in chicken growth rate, along with an increased pro-inflammatory response in caecal tissues, including upregulation of IL-17 [3]. In an attempt to assess the probiotic benefits of *L. fermentum* Biocenol CCM 7514 to a *C. jejuni* infection in vivo, we set up a pilot experiment to determine the effects of an early probiotic supplementation on body weight and cytokine expression (IL-1β, IL-15, IL-17, and IL-18) in chickens infected at 4 days of age.

## 2. Materials and Methods

### 2.1. Chickens and Experimental Design

Experiments were performed following the protocol no. 863/17-221, according to the guidelines established by the Ethical Commission of the University of Veterinary Medicine and Pharmacy in Košice, and approved by the National Veterinary and Food Administration of the Slovak Republic. A total of 108 broiler cock chickens (COBB 500) were used in the experiment, they had constant access to water and feed (free of probiotics, antibiotics, or coccidiostats) ad libitum. Individuals (27 per treatment) were allocated in four groups, following an experimental design used in previous research [20]. (i) the control group, in which neither of the bacteria were used, (ii) the probiotic group (*L. fermentum*, LB), (iii) the *Campylobacter*-challenged group (Cj), and (iv) the co-exposure group (LBCj). Within each group, chickens were subdivided into three separately-housed subgroups of nine birds, as measurements were taken at three different time points (Table 1). For sampling, three birds were weighted, sacrificed and caecal sections collected at a time, this process was performed in triplicate per time point (*n* = 9).

Animals were floor reared (9 birds/m^2^) at an ambient temperature of 30–32 °C during the entire experiment. Chickens were exposed to 24 h of continuous light for the first two days of growth, and subsequently to a regime of 23 h of light and one of dark. Environmental conditions were maintained according to the broiler breeding standards [24].

*L. fermentum* Biocenol CCM 7514 was cultivated exactly as hitherto described [20]. *C. jejuni* CCM 6189 was grown in blood-free selective agar base CM0935 supplemented with SR0155 (containing Cefoperazone and Amphotericin B; Oxoid Ltd., United Kingdom) in a jar with microaerobic conditions (5% O_2_, 10% CO_2_, 85% N_2_, CampyGen; Oxoid Ltd.). Incubation was performed for 2 days at 42 °C. *C. jejuni* colonies were harvested and diluted in PBS to the specific viable concentration (1 × 10^8^ CFU/mL). Bacterial number was controlled using optical density at 550 nm. Bacterial numbers were determined according to a standard microbiological method using serial dilution and posterior plating and prepared as previously detailed [9]. 

A *L. fermentum* suspension (10^9^ colony-forming units (CFU)/0.2 mL) was administered daily for 7 days to the LB and LBCj groups per os. On the fourth day, chickens from groups Cj and LBCj were inoculated with a suspension of *C. jejuni* (10^8^ CFU/0.2 mL). Samples were collected on day 5 (12 h post-infection (hpi)) from one of subgroup per group, on day 6 (36 hpi) and on day 7 (48 hpi) from the other two subgroups (Table 1); caecal sections were maintained in RNA-later (Thermo Scientific, Waltham, MA) and stored at –80 °C.

### 2.2. Body Weight of Chickens

The weight of each broiler chicken was recorded on a daily basis and on day 5 (12 h post-infection (hpi)), 6 (36 hpi) and 7 (48 hpi) using an analytical scale (BOECO, Germany) (Table 1).

### 2.3. RNA Extraction, Reverse Transcription, and Quantitative Polymerase Chain Reaction (PCR) Assays

Total RNA purification was performed using the RNeasy mini kit (Qiagen, Hilden, Germany) according to the instructions. Reverse transcription was carried out using iScript cDNA Synthesis Kit (Bio-Rad, Hercules, CA). The resulting cDNA was diluted in 10× in UltraPure™ DNase/RNase-Free distilled water (Invitrogen, Waltham, MA) and either employed as a template for Reverse transcription quantitative PCR (RT-qPCR) or stored at –20 °C. Primers used for determining the relative expression of IL-1β, IL-15, IL-17, and IL-18 are listed in Table 2. Amplification, detection, cycling conditions, melting curve assessment and data normalization were set as previously described [25]. Samples were analyzed in duplicate and mean values were used for subsequent analyses (Table S1). 

### 2.4. Statistical Analysis

A one-way analysis of variance, along with the Tukey post hoc test, was employed to determine the significant differences between the experimental groups. Relationships among the indicators were assessed using Pearson’s *r* correlation coefficient. Analyses were performed on MATLAB^®^ version 9.9.9341360 (MathWorks, Natick, MA, USA) (R2016a); heatmaps were obtained using Python’s plotting library, Matplotlib 3.0.3 (Python Software Foundation, DE, USA).

## 3. Results

### 3.1. Body Weight of Chickens

**Figure 1:**
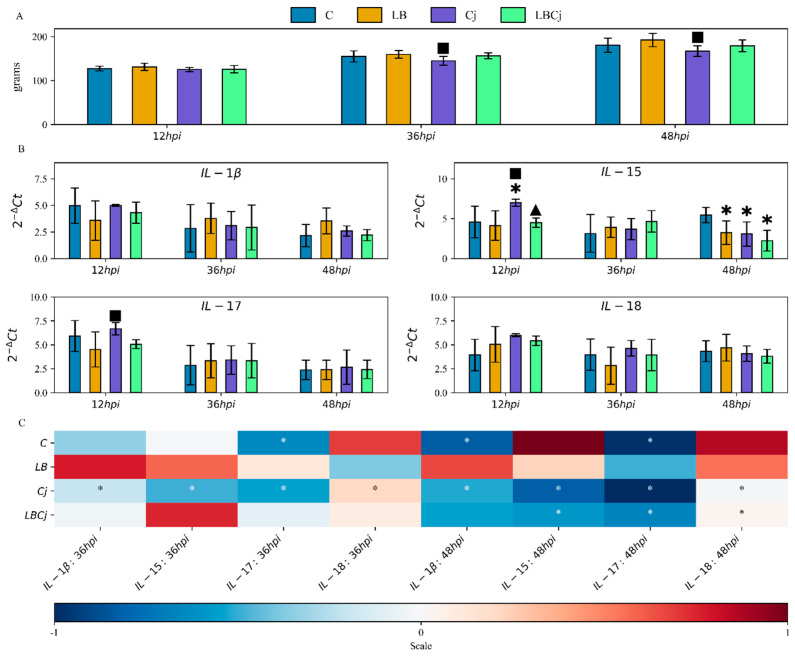
Effects on body weight and pro-inflammatory cytokine expression of *L. fermentum* and *C. jejuni* colonization at three different stages. (**A**) Average body weights of treated and untreated animals at 12, 36 and 48 hpi. (**B**) mRNA expression levels of caecal pro-inflammatory cytokines at three different time points. The Ct values of studied genes were normalized to a Ct value of the reference gene (GAPDH) (Delta -Δ- Ct), and calculated as 2^−ΔCt^. Values are mean ± standard error SE (*n* = 9). * denotes significant differences (Tukey’s test, *p* < 0.05) with the control group; ^■^ with the *L. fermentum* treatment, ^▲^ with *C. jejuni* treatment. C, control group; LB, *L. fermentum* group; Cj, *C. jejuni* group; LBcj, co-exposure group; hpi, hour post-infection. (**C**) Heat map denoting transcriptional fold change between time points. The colour scale, −1 (blue) to +1 (red), denotes mRNA levels relative to the values of the control group, with white designating down-regulated genes and black designating up-regulated genes. Relative expression was calculated and expressed as 2^−ΔΔCt^ log_2_ fold change. Values are mean ± SE (*n* = 9). * denotes significant differences (Tukey’s test, *p* < 0.05). C, control group; LB, *L. fermentum* group; Cj, *C. jejuni* group; LBcj, co-exposure group; hpi, hour post-infection.

Compared to controls conditions, consumption of *L. fermentum* increased body weight at all time points, although not significantly (*p* > 0.05). Significant differences were observed between the probiotic and pathogen groups at 36 and 48 hpi (*p* < 0.05); chickens challenged with *C. jejuni* showed an overall decrease in body weight. Infection with *C. jejuni* did not induce such a response in the presence of the probiotic group, as the average body weight of these individuals was similar to those determined in chickens not inoculated with *C. jejuni* (Figure 1A). 

### 3.2. Cytokine Expression

One-day old broiler chickens were exposed to the probiotic, and later (4 days of age) inoculated with the pathogen to investigate their effects on pro-inflammatory cytokine expression. IL-1β is mainly produced by activated macrophages and has been linked to chronic conditions, high levels of this cytokine have been observed to enhance the intensity of inflammation [29]. During the experiment, the abundance of this cytokine was altered neither by the probiotic nor by the pathogen, or the administration of both (Figure 1B) (Table S1). IL-15 modulates natural killer (NK) cells, T cell activation and proliferation, and thus is considered a key mediator of inflammation [30]. The expression of IL-15 was modified by all bacterial treatments, especially at 12 and 48 hpi. In the first stage *C. jejuni* elicited a significant upregulation compared to control conditions (Figure 1B). Similarly, mRNA levels were significantly higher in *C. jejuni*-challenged individuals than in those treated with the probiotic in the presence and absence of the pathogen (Figure 1B) (Table S1). IL-17, synthesized by T helper 17 cells (Th17), triggers the production of chemokines that attract monocytes and neutrophils. Activation of this interleukin is normally detected in the pathogenesis of various bacterial and parasitic infection [31]. *C. jejuni* colonization, if compared to *L. fermentum*, promoted an active transcription of this factor at 12 hpi (Figure 1B) (Table S1). Interleukin-18, mainly synthesized by macrophages, promotes T helper cell (Th1) development and production of IFN-γ by CD4, CD8 T cells and NK cells [32]. The present investigation revealed no significant differences in relation to this factor between treatments at any time point (Figure 1B) (Table S1). 

**Figure 2:**
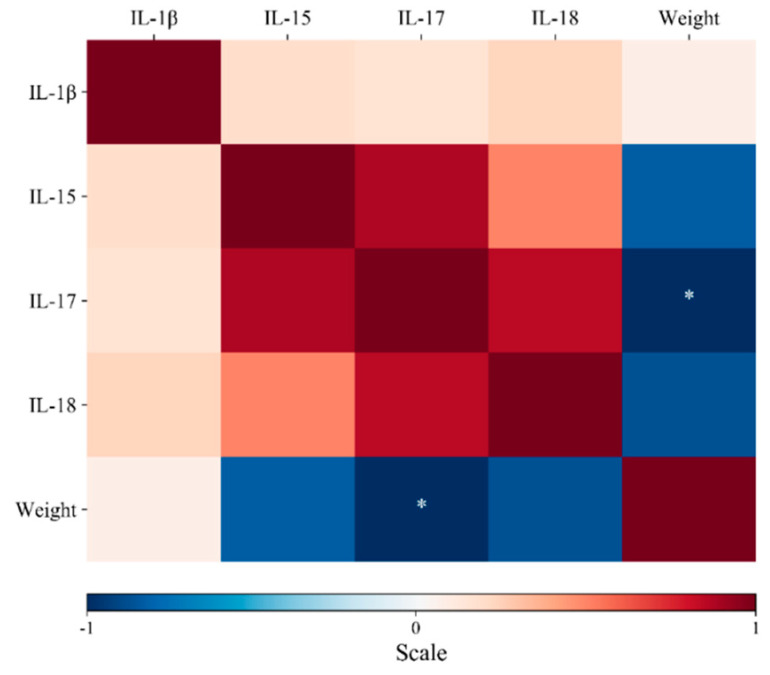
Heat map representing Pearson’s *r* correlation coefficient matrix among the indicators. Scores are denoted in color from -1 (blue) to 1 (red). * Indicates significant differences (Tukey’s test, *p* < 0.05). The negative value of the coefficient implies that IL-17 and body weight are inversely related; in other words, when this cytokine is upregulated, a loss in body weight might be expected.

As shown in Figure 1B, administration of either the probiotic or the pathogen induced a different response in gene expression at 12 hpi; such expression varied at later stages depending on the treatment. In untreated animals, for instance, IL-17 was significantly downregulated at 36 (2-fold) and 48 hpi (2.4-fold), while IL-1β was significantly downregulated only at 48 hpi (2.3-fold), respectively. IL-18 and IL-15 were upregulated, although not significantly (Figure 1C) (Table S1). In *L. fermentum*-exposed individuals, IL-15 transcript abundance increased at 36 hpi. At 48 hpi, the transcriptional profile was similar to that of the first time point; all variations in expression were nonetheless not statistically significant (Figure 1C). On the other hand, in *C. jejuni*-infected chickens, expression of IL-15 was induced at 12 hpi (Figure 1B); this factor along with the others were significantly downregulated, especially at later stages (at least 2-fold) (Figure 1C). In the co-exposure group, initial conditions involved the expression of IL-18 followed by IL-17, IL-15 and IL-1β (Figure 1B). However, at the second time point IL-15 was the most abundant, while at the third time point cytokine levels returned to those observed at 12 hpi, although significantly lower for IL-15 (2-fold), IL-17 (2.1-fold), and IL-18 (1.4-fold) (Figure 1C). Pearson’s *r* analysis revealed high positive correlations between IL-15 and IL-17, although they were not significant. On the other hand, significant negative correlations (*p* < 0.05) were also found between IL-17 and body weight (Figure 2). The negative value of the coefficient (−0.97, blue color) implies that the two variables move in different directions; namely the increased abundance of IL-17 transcripts was directly associated with the decrease in body weight observed after pathogen colonization. The raw data can be found in Table S2.

## 4. Discussion

This investigation was conceived to gain a better understanding of the effects on body weight and pro-inflammatory cytokine expression of *L. fermentum* supplementation in chickens infected with *C. jejuni*. Moreover, in order to study the consequences of post-natal probiotic treatment to an early infection, *L. fermentum* was administered from the first day of the experiment, and samples were taken 12, 36 and 48 hpi. Increased inflammation has been linked to body weight loss in pathogen-challenged chickens [33]. Early colonization by *C. jejuni* results in a transient growth rate reduction and an increased pro-inflammatory response [3]. Body weight gain was determined following challenge with *C. jejuni*, but no significant differences were detected between the control and experimental groups at any point. However, animals exposed to the probiotic showed a slight significant increase in weight compared to those infected with the pathogen. Due to the reported beneficial effects of lactic acid bacteria on bird well-being and growth [34]. and the detrimental consequences of a *C. jejuni* infection [3], it would be reasonable to expect that the infected birds would not perform as well as those fed with the probiotic. Interestingly, the weight reduction caused by the pathogen was not observed in the co-exposure group, demonstrating that the probiotic was able to lessen the impact of *C. jejuni* negative effects.

As aforementioned, overexpression of pro-inflammatory cytokines has been related to pathogenicity and weight loss [3,33]. Thus, we decided to study the effects of bacterial treatments on the expression of key pro-inflammatory cytokines. It is well documented that microorganism-associated molecular patterns (e.g., LTA—lipoteichoic acid, wall teichoic acid, peptidoglycan) of *Lactobacillus* species are responsible for activation of specific receptors in macrophages as well as epithelial and dendritic cells, which activate downstream signaling inducing cytokine production [35]. IL-1β abundance was not altered by any of the treatments during the experiment. Exposure to various *Lactobacillus* species has promoted the upregulation of this factor, although in vitro [36]. This indicates that *L. fermentum* is not as effective as other species (e.g., *L. acidophilus*, *L. reuteri*, and *L. salivarius*) at inducing the expression of pro-inflammatory cytokines. Transcript abundance of this factor has been elicited by the purified lipooligosaccharide (LOS) of *C. jejuni* HS:10, although expression was determined from spleen samples [37]. Our results suggest that IL-1β is not directly involved in the response to an *L. fermentum* treatment in *C. jejuni*-challenged chickens, especially during the first two days after pathogen infection. The expression of IL-15 was modified by all bacterial treatments; *C. jejuni,* in particular, elicited an upregulation of this cytokine compared to what was observed in the control and probiotic groups. These outcomes corroborate previous findings showing that *C. jejuni* infection increases IL-15 expression, which has been linked to cellular necrosis and pro-inflammatory responses [38]. Similarly, probiotic treatments, making use of *L. fermentum*, have shown to induce an overall reduction of IL-15 abundance [20]. Conclusively, the probiotic treatment seemed to be quite effective at downregulating IL-15 expression, which could be otherwise increased by *C. jejuni* especially at 12 hpi.

*C. jejuni* administration, if compared to *L. fermentum*, elicited the expression of IL-17 uniquely at the first time point; upregulation of this cytokine has been previously reported in response to a *C. jejuni* infection [39]. Interestingly, other *Lactobacillus* species, particularly *L. plantarum* ZS2058, activated the IL-17 pathway [39]. These data reinforce the notion that *L. fermentum* is not as efficient at inducing expression of Th17 cytokines as other species of lactic acid bacteria. Lactobacilli synbiotics, composed of *L. salivarius* and galactooligosaccharides, were shown to induce a down-regulatory pattern over cytokine expression (IL-12, IL-8, IL-1β), while levels of IL-18 were in fact upregulated by such treatment [40]. In the present research, expression levels of IL-8 were not significantly modified by any of the treatments, which differ from previous findings, indicating a decrease in mRNA abundance after *L. fermentum* administration [20]. On the other hand, these outcomes agree with previous investigations revealing that *C. jejuni* administration could not modify IL-18 levels [6]. Arguably, IL-18 does not seem to play a critical role in the response to a *C. jejuni* infection in the presence or absence of this probiotic.

Exposure to the probiotic or pathogen modulated gene expression, especially at 12 hpi. In *L. fermentum*-exposed individuals, cytokine expression was not altered, implying that the probiotic did not promote transcription of the pro-inflammatory factors. On the contrary, *C. jejuni* did modify significantly the abundance of the evaluated interleukins. In the co-exposure group, cytokine expression was observed to vary importantly throughout the experiments, especially at 36 hpi. These outcomes demonstrate that *L. fermentum* is able to stabilize expression of the studied cytokines, which even in control conditions was observed to change significantly. Moreover, the probiotic treatment was proved to induce downregulation of pro-inflammatory factors in pathogen infected chickens. Arguably, *L. fermentum* seems capable of promoting intestinal homeostasis, at least in terms of pro-inflammatory cytokine abundance. Pearson’s *r* coefficient analysis revealed a significant inverse correlation between body weight and IL-17 abundance. Early colonization by *C. jejuni* induced a decrease in chicken growth rate along with an increased pro-inflammatory response in caecal tissues, including upregulation of IL-17 [3]. IL-17 is one of the strongest inflammatory mediators and is involved in the regulation of the immune response and the development of caecal lesions by recruiting neutrophils to the site of infection [41]. In fact, antibody-mediated neutralization of IL-17 resulted in reduced caecal lesions and enhanced body weight gains in pathogen-exposed chickens [41]. Our results suggest that *L. fermentum* treatment can maintain caecal IL-17 transcription levels unaltered, which in turn could have a positive effect on gut health and ultimately body weight. Actually, the registered body weight reduction in the *C. jejuni* treatment was not observed when infection occurred after probiotic supplementation, demonstrating the benefit of using lactic acid bacteria to promote gut integrity during pathogen invasion. 

Recent experimentation by our group has revealed that levels of IL-15 and IL-17 are modulated by *L. fermentum* treatment in *C. coli* infected broilers [20]. These cytokines were downregulated after exposure to the probiotic. The low abundance of these cytokines may prevent intraepithelial lymphocytes (IEL) and memory CD8 T cells from differentiating into cytotoxic T cells, thus limiting the onset of inflammation. In fact, commensal bacteria are known to contribute to the maintenance of IELs, which are involved in wound healing and intestinal homeostasis restoration by secreting factors that promote growth [30]. Previous research has shown that *Lactobacillus* spp. administration reduces chicken pro-inflammatory cytokine expression and improves weight gain [34]. Here, we have revealed that *L. fermentum* dampens expression of pro-inflammatory factors and provides a slight advantage on weight gain in *C. jejuni*-challenged birds. The current results corroborate the above-mentioned findings, and emphasize the fact that IL-15 and IL-17 are key players in the response to an early *Campylobacter* spp. infection in the presence of the probiotic. Upregulation of these cytokines was elicited mainly by the presence of *C. jejuni*. Recent research has demonstrated that the abundance IL-1β and IL-18 increased significantly as a response to an early chicken infection by *C. jejuni* in the presence of *L. reuteri* [8]. The secretion of these factors is known to occur in response to bacterial infection or cellular damage, as they are key modulators of the initiation of inflammation [21]. The present outcomes showed that these cytokines are not upregulated by *L. fermentum*, which support the notion that this lactic acid bacteria have a mild effect, in terms of promoting pro-inflammatory cytokine expression, compared to other species of *Lactobacillus* [8,19].

The immune response to *Campylobacter* infection in chickens is complex, and starts with bacterial recognition by pattern recognition receptors (PRR), which upon activation promote expression of pro-inflammatory cytokines [42]. The present results show that the abundance of some these cytokines increased at 12 hpi, but later decreased at 48 hpi. This decline after the initial response has been reported previously after *Campylobacter* colonization [39], which implies that the host immune system deals with the initial colonization as an attack, although it later reaches a certain level of tolerance [43,44]. Arguably, chickens mount an immune response to infection by *Campylobacter*. Despite the fact that the initial response seems no different from those elicited by commensal organisms, various reports of pathology have demonstrated that *Campylobacter* might trigger a diseased-like state [7,41], which suggest that *C. jejuni* should not be regarded solely as commensal but instead as a pathogen [2].

## 5. Conclusions

The data presented herein highlight the benefits of utilizing probiotics for avoiding the negative influences of *Campylobacter* spp. infection on bird body weight and inflammation. However, further experiments must explore these effects at even later stages, and must include other cytokines (e.g., IL-12, IL-22, and IFN-γ), especially Th2 cytokines such as IL-4, IL-6 or IL-10. In addition, the expression of other inflammatory mediators, especially those involved in the prostaglandin signaling pathway, must be assessed in order to gain a better understanding of *Campylobacter*-induced intestinal inflammation and its modulation by lactic acid bacteria.

## Figures and Tables

**Table 1:** Schema of the methodology aimed at measuring cytokine transcript modulation in chickens exposed to *L. fermentum* and *C. jejuni*.

Day of Experiment	Control (number of chickens)	LB Group (number of chickens)	Cj Group (number of chickens)	LBCj Group (number of chickens)
0 d	27	27	27	27
1 d	27	27 *L. fermentum* dose 10^9^ CFU/0.2 mL individually *per os*	27	27 *L. fermentum* dose 10^9^ CFU/0.2 mL individually *per os*
2 d	27	27 *L. fermentum* dose 10^9^ CFU/0.2 mL individually *per os*	27	27 *L. fermentum* dose 10^9^ CFU/0.2 mL individually *per os*
3 d	27	27 *L. fermentum* dose 10^9^ CFU/0.2 mL individually *per os*	27	27 *L. fermentum* dose 10^9^ CFU/0.2 mL individually *per os*
4 d	27	27 *L. fermentum* dose 10^9^ CFU/0.2 mL individually *per os*	27 *C. jejuni* dose 10^8^ CFU/0.2 mL individually *per os*	27 *L. fermentum* dose 10^9^ CFU/0.2 mL + *C. jejuni* dose 10^8^ CFU/0.2 mL individually *per os*
5 d (12 hpi) sample collection	18	18 *L. fermentum* dose 10^9^ CFU/0.2 mL individually *per os*	18	18 *L. fermentum* dose 10^9^ CFU/0.2 mL individually *per os*
6d (36 hpi) sample collection	9	9 *L. fermentum* dose 10^9^ CFU/0.2 mL individually *per os*	9	9 *L. fermentum* dose 10^9^ CFU/0.2 mL individually *per os*
7d (48 hpi) sample collection	9	9 *L. fermentum* dose 10^9^ CFU/0.2 mL individually *per os*	9	9 *L. fermentum* dose 10^9^ CFU/0.2 mL individually *per os*

C, Control group; LB, *Lactobacillus fermentum*; Cj, *Campylobacter jejuni*; hpi, hours post-infection.

**Table 2:** List of primers utilized in RT-qPCR for cytokine transcript detection.

Primer	Sequence 5′-3′	Reference
IL-1β For IL-1β Rev	GAAGTGCTTCGTGCTGGAGT ACTGGCATCTGCCCAGTTC	[26]
IL-15 For IL-15 Rev	TGGAGCTGATCAAGACATCTG CATTACAGGTTCCTGGCATTC	[27]
IL-17 For IL-17 Rev	TATCAGCAAACGCTCACTGG AGTTCACGCACCTGGAATG	[26]
IL-18 For IL-18 Rev	ACGTGGCAGCTTTTGAAGAT GCGGTGGTTTTGTAACAGTG	[25]
GAPDH For GAPDH Rev	CCTGCATCTGCCCATTT GGCACGCCATCACTATC	[28]

## Supplementary Materials

The following are available online at https://www.mdpi.com/2306-7381/7/3/121/s1, Table S1: Relative mRNA expression of caecal pro-inflammatory cytokines in probiotic and pathogen-colonized chickens, Table S2: Raw data.
