# Supplementary material for: Effects of Lactobacillus Fermentum Supplementation on Body Weight and Pro-Inflammatory Cytokine Expression in Campylobacter Jejuni-Challenged Chickens
Source: Vet Sci. 2020 Aug 29;7(3):121. doi: 10.3390/vetsci7030121 (PMC7557755; doi:10.3390/vetsci7030121)
Supplement: Supplementary file 1 [file vetsci-07-00121-s001.zip › vetsci-918767-supplementary - final-1/vetsci-918767-TableS1.docx]

**Table 1.** Relative mRNA expression of caecal pro-inflammatory cytokines in probiotic and pathogen-colonized chickens

| Expression levels (2^–Δ^Ct) | | | | | | | | | | | | |
| --- | --- | --- | --- | --- | --- | --- | --- | --- | --- | --- | --- | --- |
|  | **12 hpi** | | | | **36 hpi** | | | | **48 hpi** | | | |
| **Gene symbol** | **Control** | ***L. fermentum*** | ***C. jejuni*** | ***L. fermentum* + *C. jejuni*** | **Control** | ***L. fermentum*** | ***C. jejuni*** | *L. fermentum* + *C. jejuni* | Control | *L. fermentum* | *C. jejuni* | *L. fermentum* + *C. jejuni* |
| IL-1β | 4.96 ± 1.69^a^ | 3.58 ± 1.85^a^ | 4.99 ± 0.09^a^ | 4.31 ± 1.01^a^ | 2.84 ± 2.22^b^ | 3.78 ± 1.43^a^ | 3.08 ± 1.33^b^ | 2.92 ± 2.12^a^ | 2.17 ± 1.05^b^ | 3.54 ± 1.21^a^ | 2.60 ± 0.48^b^ | 2.21 ± 0.52^a^ |
| IL-15 | 4.58 ± 1.98^a^ | 4.14 ± 1.85^▲a^ | 7.01 ± 0.45*^a^ | ­­4.49 ± 0.59^▲a^ | 3.14 ± 2.36^a^ | 3.92 ± 1.27^a^ | 3.70 ± 1.32^b^ | 4.65 ± 1.35^a^ | 5.45 ± 0.94^a^ | 3.25 ± 1.49*^a^ | 3.08 ± 1.52*^b^ | 2.25 ± 1.31*^b^ |
| IL-17 | 5.91 ±1.62^a^ | 4.52 ± 1.83^▲a^ | 6.67 ± 0.63^a^ | 5.06 ± 0.46^a^ | 2.86 ± 2.05^b^ | 3.32 ± 1.77^b^ | 3.41 ± 1.48^b^ | 3.33 ± 1.80^b^ | 2.38 ± 1.01^b^ | 2.39 ± 1.01^b^ | 2.67 ± 1.79^b^ | 2.42 ± 0.97^b^ |
| IL-18 | 3.93 ± 1.64^a^ | 5.04 ± 1.87^a^ | 5.99 ± 0.16^a^ | 5.43 ± 0.50^a^ | 3.96± 1.64^a^ | 2.82 ± 1.94^b^ | 4.63 ± 0.81^b^ | 3.92 ± 1.64^b^ | 4.33 ± 1.09^a^ | 4.70 ± 1.39^a^ | 4.54 ± 1.14^b^ | 3.80 ± 0.71^b^ |

Values are represented as mean ±SD (n = 9). The Ct values of studied genes were normalized to a Ct value of the reference gene (GAPDH) (^Δ^Ct), and calculated as 2–^Δ^Ct.

At each time point: * denotes significant differences (Tukey’s test, *p* < 0.05) with control group; ^▲^ with *C. jejuni* treatment.

Different superscript letters (a, b) show significant differences (*p* < 0.05), between time points, of each experimental group.

hpi, hours post-infection; IL, interleukin; GADPH, glyceraldehyde-3-phosphate dehydrogenase.
